# Specimen specific imaging and joint mechanical testing data for next generation virtual knees

**DOI:** 10.1016/j.dib.2021.106824

**Published:** 2021-01-30

**Authors:** Snehal Chokhandre, Erica E. Neumann, Tara F. Nagle, Robb W. Colbrunn, Chris A. Flask, Ceylan Colak, Jason Halloran, Ahmet Erdemir

**Affiliations:** a Department of Biomedical Engineering, Lerner Research Institute, Cleveland Clinic, Cleveland, OH, United States; b Computational Biomodeling (CoBi) Core, Lerner Research Institute, Cleveland Clinic, Cleveland, Ohio, United States; c BioRobotics and Mechanical Testing Core, Lerner Research Institute, Cleveland Clinic, Cleveland, Ohio, United States; d Department of Radiology, Case Western Reserve University, Cleveland, Ohio, United States; e Department of Biomedical Engineering, Case Western Reserve University, Cleveland, Ohio, United States; f Department of Pediatrics, Case Western Reserve University, Cleveland, Ohio, United States; g Department of Diagnostic Radiology, Cleveland Clinic, Cleveland, Ohio, United States; h Institute for Shock Physics, Washington State University, Pullman, WA, United States

**Keywords:** Human knee, Finite element model, MRI, Mechanics, Specimen-specific

## Abstract

Virtual knees, with specimen-specific anatomy and mechanics, require heterogeneous data collected on the same knee. Specimen-specific data such as the specimen geometry, physiological joint kinematics-kinetics and contact mechanics are necessary in the development of virtual knee specimens for clinical and scientific simulations. These data are also required to capture or evaluate the predictive capacity of the model to represent joint and tissue mechanical response. This document details the collection of magnetic resonance imaging data and, tibiofemoral joint and patellofemoral joint mechanical testing data*.* These data were acquired for a cohort of eight knee specimens representing different populations with varying gender, age and perceived health of the joint. These data were collected as part of the Open Knee(s) initiative. Imaging data when combined with joint mechanics data, may enable development and assessment of authentic specimen-specific finite element models of the knee. The data may also guide prospective studies for association of anatomical and biomechanical markers in a specimen-specific manner.

## Specifications Table

**Table:** 

Subject	Bioengineering
Specific subject area	Finite element modeling of joints
Type of data	Magnetic resonance imaging (.nii files) Mechanical testing data (raw, .tdms files) Figures Tables Python scripts
How data were acquired	MRI raw data (Siemens Skyra, 3.0 Tesla human MRI scanner, Siemens Healthneers Malvern, PA, USA), with a knee receiver coil) Tibiofemoral joint testing data (6 DoF parallel robot; Rotopod R-2000, Mikrolar, Hampton, NH, USA) Patellofemoral joint testing data (6 DoF parallel robot; Rotopod R-2000, Mikrolar, Hampton, NH, USA), Tekscan pressure sensor 5051, 8.27 MPa range(Tekscan Inc, Boston, MA, USA)
Data format	MRI images (.nii) Joint mechanical testing data (.tdms) Pressure data (.csv)
Parameters for data collection	Healthy body mass index (BMI), no injury or surgery at the joint
Description of data collection	Imaging and joint mechanical testing data for cadaveric human knee joints
Data source location	Cleveland Clinic Cleveland, OH USA
Data accessibility	Data publicly accessible at, https://doi.org/10.18735/4e78–1311 (2020)

## Value of the Data

• The data set provides specimen specific anatomical and mechanical characterization information for human knees in details that were not publicly available before. The data set was built to aid development of virtual knees that can be built and validated using the actual specimen specific information.
• As the time and resources required to obtain these comprehensive data can be prohibitive for many researchers, public availability of such data can help researchers bypass the efforts required to obtain them and utilize the data or parts of them to address their research interests in knee mechanics.
• Mechanical data can be used for understanding knee joint mechanics and imaging data can be leveraged for image analysis. General purpose models built and validated using these data can be used for various purposes such as training, aiding clinical decision making, virtual implant testing, conducting virtual experiments to explore joint and tissue mechanics etc. Publicly accessible detailed specifications can be used to obtain similar data for additional specimens or improve upon existing protocols and specifications.

## 1 Data Description

The Open Knee(s) project provides data for eight cadaver knee specimens. The target sample population was male and female, young (18–35 years) with healthy cartilage, middle aged (40–65 years) with healthy cartilage, elderly (65–80 years) with a mix of healthy and osteoarthritic cartilage. Other requirements of the individuals from which specimens were obtained included: height (1.5–1.8 m), weight (45–90 kg), and Body Mass Index (BMI, 18.5–24.9 considered to be the normal range). Prior to specimen acquisition, X-rays provided by specimen vendors were evaluated by surgeons to assess overall joint health to decide suitability of the specimen for the study. This assessment provided us with confirmation that the knee had no indication of injury or surgery. Specimen-specific details are provided in Table 1.

**Table 1:** Specimen properties and demographics.

Specimen ID	oks001	oks002	oks003	oks004	oks006	oks007	oks008	oks009
Side	right	right	left	right	right	right	right	left
Gender	male	female	female	female	female	male	male	male
Age (years)	71	67	25	46	71	71	40	34
Height (m)	1.83	1.55	1.73	1.58	1.52	1.70	1.78	1.80
Weight (kg)	77.1	45.3	68.0	54.4	49.4	65.8	63.5	68.03
BMI	23.1	18.9	22.8	21.9	21.3	22.7	20.09	20.0

Open Knee(s) is a free and open source modeling project primarily focusing on finite element analysis of the knee (https://simtk.org/projects/openknee). This resource curates experimentation and modeling specifications along with an evolving amount of specimen-specific data and models, and various data analysis and modeling scripts. The provided dataset is an outcome of Open Knee(s) activities; a static version of the data is accessible through the data site [1].

Images were originally acquired in the standard DICOM format, with each two dimensional (2D) image slice comprising the three dimensional (3D) volume stored in separate files. 3D Slicer (https://www.slicer.org/) and SimpleITK (http://www.simpleitk.org/) were used to simplify management and distribution of MR images by combining associated sets of corresponding 2D slices into a single file of the Neuroimaging Informatics Technology Initiative format (NIfTI, .nii, https://nifti.nimh.nih.gov/). Table 2 provides detailed file descriptions of the specimen-specific image sets with imaging protocol and image set labels.

**Table 2:** MRI data file descriptions. File names for each of the MRI set in .NII format for every specimen is provided.

Specimen ID	MRI Protocol	Filename (Filetype: NIfTI, .nii)
oks001	General Purpose	1.3.12.2.1107.5.2.19.45406.2014100710220888421542662.0.0.0.nii
	Cartilage	1.3.12.2.1107.5.2.19.45406.2014100710433217692143626.0.0.0.nii
	Connective (axial)	1.3.12.2.1107.5.2.19.45406.2014100711193292568244326.0.0.0.nii
	Connective (sagittal)	1.3.12.2.1107.5.2.19.45406.2014100711262396541244530.0.0.0.nii
	Connective (coronal)	1.3.12.2.1107.5.2.19.45406.2014100711323578731244734.0.0.0.nii
oks002	General Purpose	0003.0320.2014.11.04.11.20.53.124440.359899718.nii
	Cartilage	0004.0224.2014.11.04.11.20.53.124440.359906184.nii
	Connective (axial)	0007.0001.2014.11.04.11.20.53.124440.359915938.nii
	Connective (sagittal)	0005.0050.2014.11.04.11.22.47.588987.359909764.nii
	Connective (coronal)	0006.0001.2014.11.04.11.20.53.124440.359913880.nii
oks003	General Purpose	1.3.12.2.1107.5.2.19.45406.2014120210113013368841431.0.0.0.nii
	Cartilage	1.3.12.2.1107.5.2.19.45406.2014120210325342222042395.0.0.0.nii
	Connective (axial)	1.3.12.2.1107.5.2.19.45406.2014120211145041394943484.0.0.0.nii
	Connective (sagittal)	1.3.12.2.1107.5.2.19.45406.2014120211045428131243076.0.0.0.nii
	Connective (coronal)	1.3.12.2.1107.5.2.19.45406.2014120211095362283243280.0.0.0.nii
oks004	General Purpose	1.3.12.2.1107.5.2.19.45406.2014072210222363865584064.0.0.0.nii
	Cartilage	1.3.12.2.1107.5.2.19.45406.2014072211413113536586664.0.0.0.nii
	Connective (axial)	1.3.12.2.1107.5.2.19.45406.2014072211351581496185970.0.0.0.nii
	Connective (sagittal)	1.3.12.2.1107.5.2.19.45406.2014072211404233708286174.0.0.0.nii
	Connective (repeated sagittal)	1.3.12.2.1107.5.2.19.45406.2014072212133281316387345.0.0.0.nii
oks006	General Purpose	1.3.12.2.1107.5.2.19.45406.2015010611354553104280874.0.0.0.nii
	Cartilage	1.3.12.2.1107.5.2.19.45406.2015010611570880894681838.0.0.0.nii
	Connective (axial)	1.3.12.2.1107.5.2.19.45406.2015010612350885610582739.0.0.0.nii
	Connective (sagittal)	1.3.12.2.1107.5.2.19.45406.2015010612301630598082535.0.0.0.nii
	Connective (coronal)	1.3.12.2.1107.5.2.19.45406.2015010612400137121082943.0.0.0.nii
oks007	General Purpose	1.3.12.2.1107.5.2.19.45406.2015020310331641962506155.0.0.0.nii
	Cartilage	1.3.12.2.1107.5.2.19.45406.2015020310560389569607119.0.0.0.nii
	Connective (axial)	1.3.12.2.1107.5.2.19.45406.2015020311391094492308224.0.0.0.nii
	Connective (sagittal)	1.3.12.2.1107.5.2.19.45406.2015020311292575600307816.0.0.0.nii
	Connective (coronal)	1.3.12.2.1107.5.2.19.45406.2015020311341828039508020.0.0.0.nii
oks008	General Purpose	1.3.12.2.1107.5.2.19.45406.2015060311163837990581829.0.0.0.nii
	Cartilage	1.3.12.2.1107.5.2.19.45406.2015060311391849809482793.0.0.0.nii
	Connective (axial)	1.3.12.2.1107.5.2.19.45406.2015060312162325167483774.0.0.0.nii
	Connective (sagittal)	1.3.12.2.1107.5.2.19.45406.2015060312064169652083468.0.0.0.nii
	Connective (coronal)	1.3.12.2.1107.5.2.19.45406.2015060312113066906083621.0.0.0.nii
oks009	General Purpose	1.3.12.2.1107.5.2.19.45406.2015111710293432651200476.0.0.0.nii
	Cartilage	1.3.12.2.1107.5.2.19.45406.2015111710505772215301440.0.0.0.nii
	Connective (axial)	1.3.12.2.1107.5.2.19.45406.2015111711282322237402453.0.0.0.nii
	Connective (sagittal)	1.3.12.2.1107.5.2.19.45406.2015111711182762540902131.0.0.0.nii
	Connective (coronal)	1.3.12.2.1107.5.2.19.45406.2015111711232022410402284.0.0.0.nii

Joint mechanics data set was obtained for both tibiofemoral and patellofemoral testing and was organized in separate folders for each specimen (Appendix A1). The folder for tibiofemoral joint contains kinematics-kinetics data (in technical data management solution (TDMS) file format, http://www.ni.com/product-documentation/3727/en/, contents described in Appendix A2, Table A1) and configuration subfolders (contents of configuration files are detailed in Appendix A2, Table A2). The folder for patellofemoral joint contains subfolders for kinematics-kinetics data and contact pressures. For both test sets, the configuration folder contains information on anatomical landmarks, registration marker locations, coordinate systems, etc. (Appendix A2). It should be noted that data on patella registration marker assembly can be found in the tibiofemoral joint kinematics-kinetics folder (folder description provided in appendix A1 and A3) as the data were collected at the specimen preparation session immediately before tibiofemoral joint testing. These data include a file (CAD_PT_DIMENSIONS.txt) indicating location of points on the 3D printed MRI compatible patella registration marker assembly in CAD. This file can also be found in the ‘doc’ folder at the data site [1] (folder description provided in appendix A3). All joint testing files, including raw and processed kinematics-kinetics data, coordinate transformation matrices, contact pressure measurements, etc. are also disseminated at the data site [1].

The data site [1] contains a static copy of the data created at the time of publication. All open source code used for obtaining all necessary transformations, data analysis and visualization for both the joint mechanical testing data and the pressure data are disseminated at data site [1] (available in ‘src’ folder). The instructions for their usage are also provided through the data site [1] in the readme.txt file. Readers are also encouraged to check Open Knee(s) project site (https://simtk.org/projects/openknee) for synergistic information: experimentation and modeling specifications, an evolving amount of data and models from prospective specimens, updated data analysis and modeling scripts, a growing amount of derivative data such as geometries, processed data etc. obtained from these data sets.

### 1.1 Data validation – MRI

**Fig. 1:**
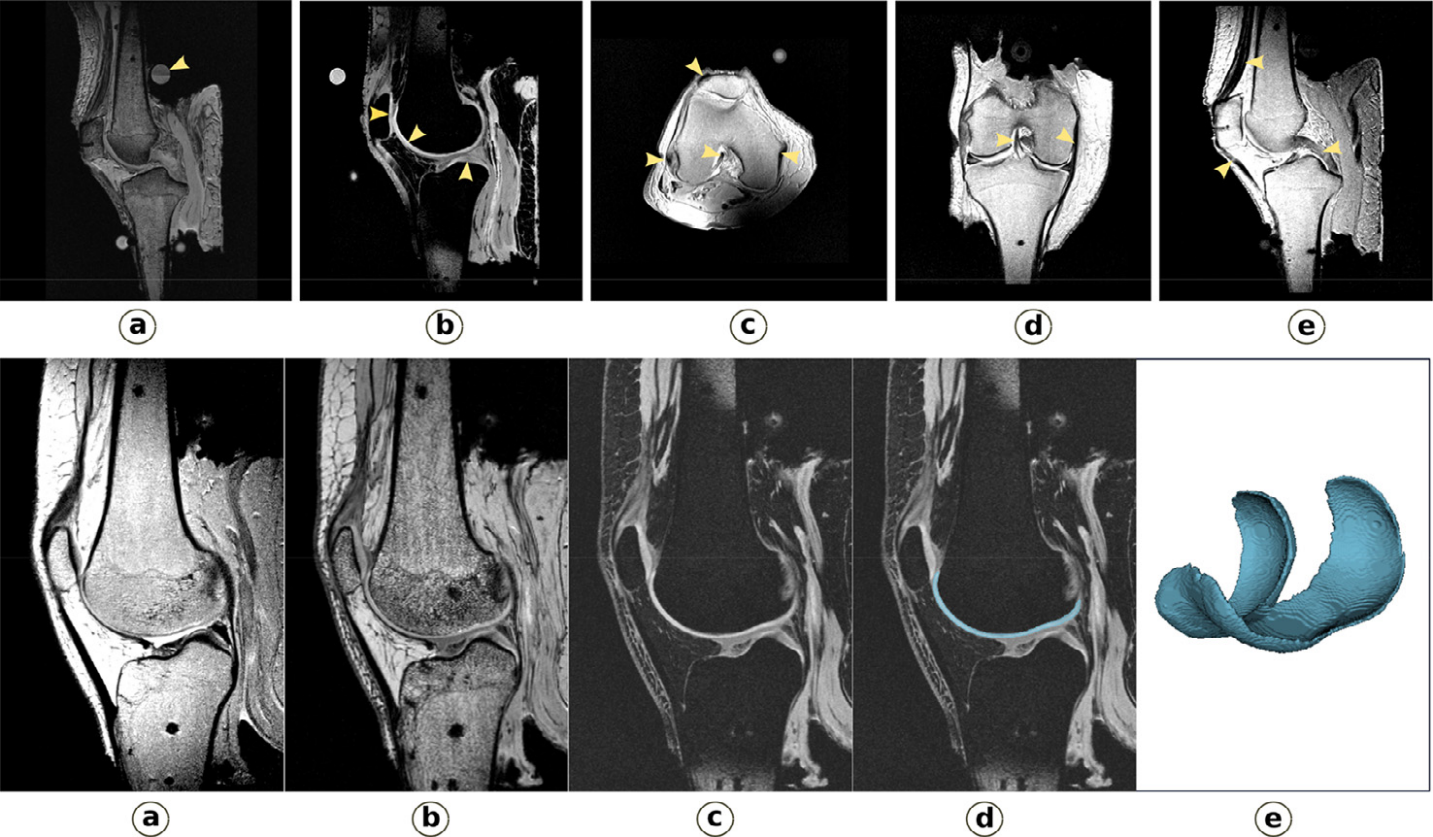
Upper row: Tissue specific magnetic resonance imaging: a) 3D, T1-weighted suitable for overall view of the joint (registration marker highlighted), b) T1-weighted sagittal with fat suppression (cartilage specific, highlighted), c)-e) Proton density, turbo-spin echo (axial, coronal and sagittal sections; connective tissue specific, highlighted). Bottom row: Segmentation of femoral cartilage for specimen oks003 a), b), and c) show the sagittal view in MRI under different protocols with (c) T1- weighted sagittal with fat suppression being the best suited for cartilage delineation. d) shows the segmentation overlaid on image and e) shows the 3D segmented femoral cartilage geometry. Also refer to Table 2 for specific imaging sequences for each specimen.

As five sets of images were acquired to target specific structures, a preliminary comparison was performed to assess their adequacy. Fig. 1 provides samples of the three MRI settings, which highlights contrasting results between all three. To demonstrate use of the cartilage focused MR settings, a manual image segmentation (Fig. 1) was performed using *3D Slicer*. User evaluation of the image sets indicated the data set's sufficiency to delineate the boundaries of tissues of interest. It is important to note that these protocols may not be appropriate for in vivo model development as the scans take more than an hour to complete. Specimen oks002 was inserted foot first in the scanner resulting in inverted images. In addition, the coronal image set for ligament specific scans is missing for oks004 (a repeated sagittal image set was accidentally acquired instead).

**Fig. 2:**
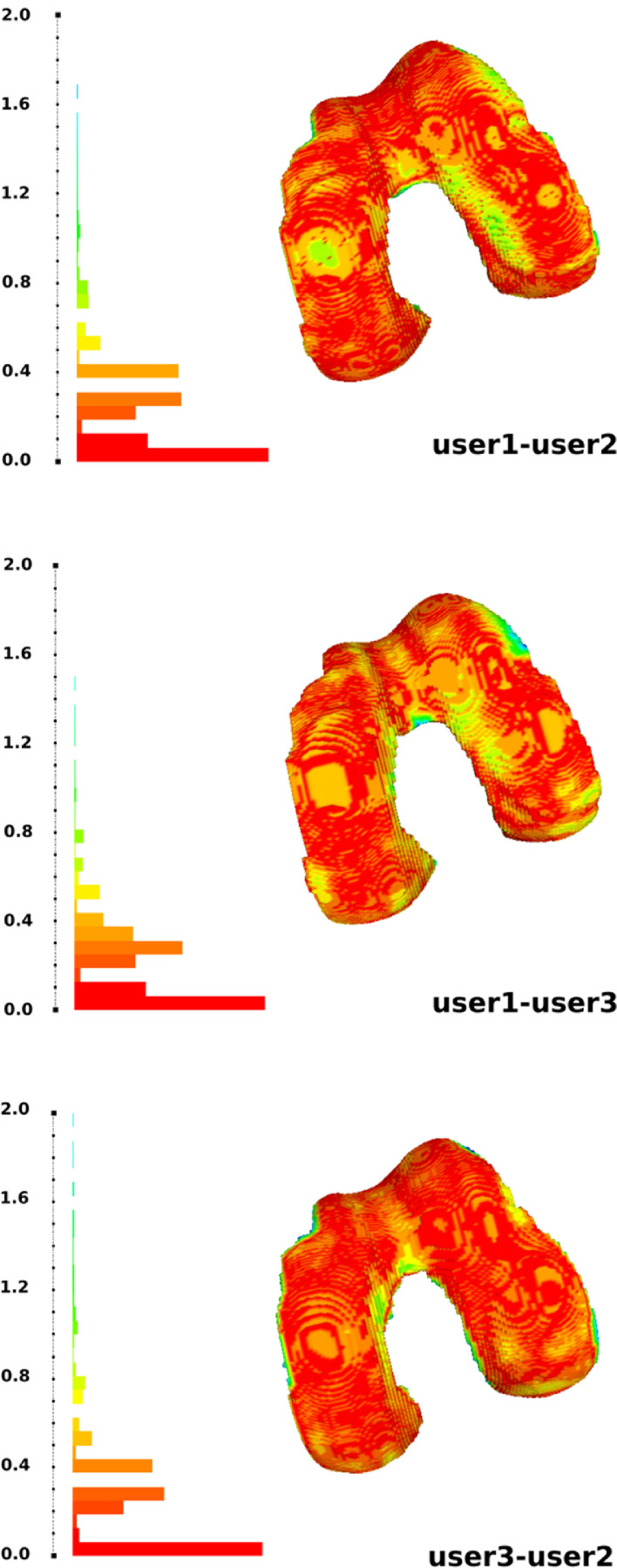
A comparison of raw segmentation of femur cartilage for specimen oks003 performed by three users. The users had varying degree of experience with manual image segmentation. The colormap shows the Hausdorff distances between the segmentation attempts.

Fig. 2 provides a sample comparison of inter-user segmentation (boundary delineation) for femoral cartilage for oks003. All segmentations were performed manually using 3D Slicer. Raw segmentations were exported as three-dimensional surfaces. Using MeshLab (https://www.meshlab.net/), Housdorff's distance was calculated between each set of attempts as a metric of variability between attempts. Users had various degrees of experience in identification of structures and using the software. Some areas of load bearing regions appear to have ∼ 0.6 mm difference. The image resolution was 0.35 mm which may explain part of the variation. Most of the load bearing region has low variation which indicates that the imaging data may be adequate for identification of structures to be modeled. More automated methods may reduce the variability in segmentation. The protocols were developed using ease of segmentation as a primary qualifier and other parameters such as signal to noise ratio, contrast to noise ratio etc. were not evaluated. Hence, the protocol evaluation was limited.

To evaluate the adequacy of the data set in ascertaining tissue quality, the image sets were assessed by a trained radiologist (completed residency abroad and had three years of experience working at musculoskeletal radiology at the Cleveland Clinic at the time of assessment). MRI Osteoarthritis Knee Score (MOAKS) [2], which is one of the most commonly used semi-quantitative MRI scoring system was used. A total of 14 sub-regions were graded to assess the cartilage, with each sub-region individually graded for size of any cartilage loss (partial or full-thickness loss), amount of cartilage loss as a percentage of sub-region area, and amount of full-thickness cartilage loss as a percentage of the sub-region. Following are the details of grading used. *(a) MOAKS: Any cartilage loss (surface area):* Cartilage: 0= none, 1= 〈 10% of region of surface area, 2 = 10 - 75%, 3= 〉 75%. (b)*MOAKS Osteophytes:* Grade 0= none; Grade 1=small; Grade 2= medium; Grade 3= large*. (c) MOAKS: Bone Marrow Lesion:* 0= none, 1= 〈 33% of sub-regional volume, 2 = 33 - 66%, 3= 〉 66%. (MOAKS femur regions: medial posterior, medial central, lateral posterior, lateral central, medial trochlea, lateral torchlea; MOAKS tibia regions: posterior medial, central medial, anterior medial, lateral posterior, lateral central, lateral anterior; MOAKS patella regions: medial, lateral). (d) *Effusion/synovitis:* Grade 0: None Grade 1: Small, fluid in the retropatellar space, Grade 2: Medium, slight convexity of the suprapatellar bursa, Grade 3: Large, evidence of capsular distention. (e*) MOAKS Meniscal morphology: N*=Normal, Deg = Degenerative signal change, Long.-rad. tear= Longitudinal-radial tear (including BH), Horizon. Tear =Horizontal tear, Comp. tear=Complex tear (horizontal+radial tear), Partial mac. = Partial maceration, Prog. part. mac. = Progressive partial maceration, Compt. mac. = Complete maceration, Menis. Cyst = Meniscal cyst, Menis. hyper. = Meniscal hypertrophy. (f) *MOAKS meniscal extrusion:* Grade 0: < 2 mm Grade 1: 2–2.9 mm Grade 2: 3–4.9 mm Grade 3: >5 mm (According to clinical grading Grade 2–3 = Positive for Extrusion). (g) *Ligaments/ Ligament related pathology:* region of interest.

Bone marrow lesions (BMLs) including edema and cyst, of 15 sub-regions (the subspinous region was added to cartilage sub-regions for BMLs) were assessed for the size as a percentage volume. The osteophytes were graded in each of the 12 locations based on size using the scale from none to large. Since the MRI were obtained postmortem, synovitis, bone marrow edema and effusion could not be graded exactly but a suboptimal evaluation was provided. Radiological examination also included gross evaluation of ligament and meniscus tissue for apparent damage. A detailed examination was not possible due to large field of view. For further details see Table 3. In order to develop accurate virtual knees for clinical and scientific simulations to design predictive models, assessment of the original structures such as cartilage damage is important. Previous studies showed that MOAKS is a useful scoring system to predict the clinical outcomes in predictive models. We believe this grading system can assess the knee more accurately for the virtual knee models [3,4].

**Table 3:** Tissue quality analysis as provided by a radiologist. See manuscript text for details of scoring systems.

Specimen	Cartilage damage	Osteophytes	Bone marrow lesions/ abnormality	Effusion	Synovitis	Meniscus morphology	Meniscal extrusion	Ligament / other soft tissuedamage
oks001	Medial patella – grade 2	Grade 0	Medial patella – grade 1	Grade 0	Grade 0	normal	Grade 0	Bone marrow abnormality at PCL Insertion
oks002	Medial central femur – grade 1 Medial trochlea – grade 1 Lateral patella – grade 2	Patella superior – grade 1	Lateral patella – grade 2	Grade 0	Grade 0	Medial posterior horn, lateral anterior horn, lateral body, lateral posterior horn - deg	Grade 0	None
oks003	Grade 0	Grade 0	Grade 0	Grade 0	Grade 0	Normal	Grade 0	None
oks004	Grade 0	Grade 0	All - Grade 3	Grade 0	Grade 0	Normal	Grade 0	None
oks006	Posterior medial tibia – grade 1 Medial patella – grade 3	Grade 0	Posterior medial tibia – grade 1 Medial patella – grade 3	Grade 0	Grade 0	Lateral anterior horn, lateral posterior horn, lateral body - deg	Grade 0	None
oks007	Lateral posterior femur – grade 1 Medial trochlea – grade 1 Medial/lateral patella – grade 2	Grade 0	Grade 0	Grade 0	Grade 0	Medial posterior horn, lateral posterior horn - deg	Grade 0	None
oks008	Grade 0	Grade 0	Grade 0	Grade 0	Grade 0	Normal	Grade 0	None
oks009	Lateral posterior femur – grade 1	Grade 0	Lateral posterior femur – grade 1	Grade 0	Grade 0	Normal	Grade 0	None

### 1.2 Data validation – joint mechanical testing

Estimates of registration errors between experiment and imaging were calculated using MRI opaque spherical marker sets on femur, tibia and patella. These spheres were reconstructed from the digitized points from experimentation and centres were calculated. Similarly, spheres were reconstructed from MRI segmentation and centres for those are also calculated. For each rigid body/bone, the distances between markers were calculated from both experimentation and imaging. The difference between distances obtained from experimentation and imaging were then calculated. An average of differences in distances between experimentation and imaging were defined as registration errors. Average registration errors are reported in Table 4.

**Table 4:** Estimated registration errors. Errors indicate the distance deviations between registration marker locations as measured by joint testing and by imaging. Percentage errors (in brackets) represent error magnitude relative to total distance between markers. Patella errors were not reported for specimen oks004 due to missing patella registration marker assembly.

Specimen	Femur	Tibia	Patella
oks001	1.95 mm [3.63%]	1.07 mm [1.93%]	0.66 mm [1.09%]
oks002	1.06 mm [2.27%]	0.96 mm [1.61%]	0.74 mm [1.21%]
oks003	0.65 mm [1.24%]	0.32 mm [0.55%]	0.63 mm [1.05%]
oks004	0.90 mm [2.41%]	0.61 mm [1.38%]	–
oks006	1.42 mm [3.52%]	0.34 mm [0.70%]	0.67 mm [1.12%]
oks007	1.69 mm [3.64%]	0.68 mm [1.44%]	0.54 mm [0.90%]
oks008	0.83 mm [1.69%]	0.56 mm [0.87%]	0.44 mm [0.74%]
oks009	1.57 mm [3.38%]	0.12 mm [0.26%]	0.59 mm [1.00%]

**Fig. 3:**
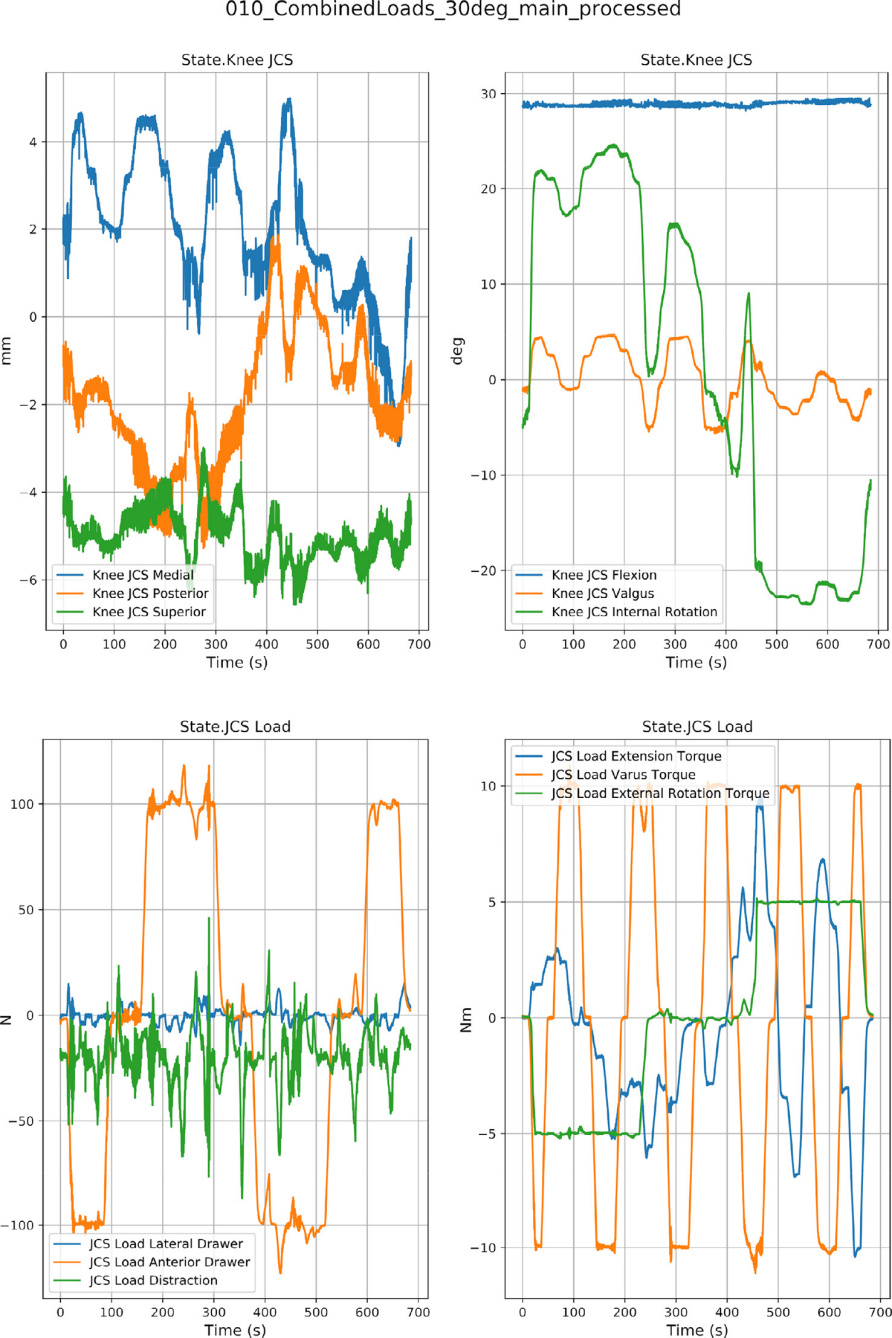
An example data set from combined loading test. Data are shown for specimen oks003 at 30° flexion. Actual kinematics (Channel: State.Knee JCS) and Actual kinetics (Channel: State.JCS Load).

**Fig. 4:**
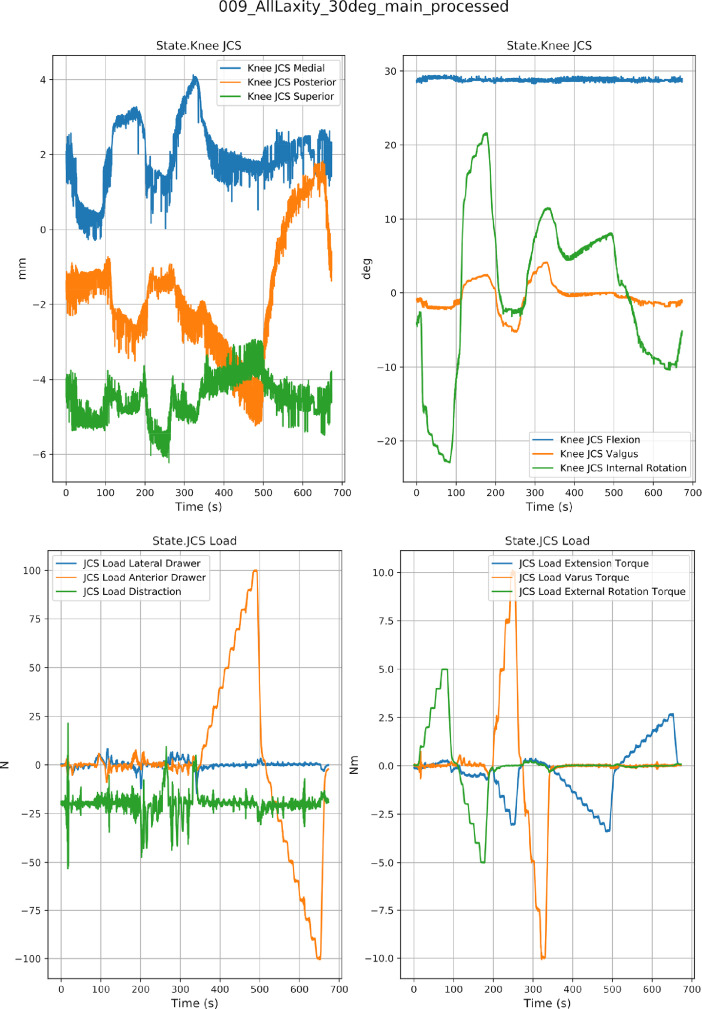
An example data set from laxity testing. Data are shown for specimen oks003 at 30° flexion. Actual kinematics (Channel: State.Knee JCS) and Actual kinetics (Channel: State.JCS Load).

Fig. 3, Fig. 4 show the raw combined loading and laxity data for specimen oks003, respectively. Python scripts were developed to down sample the data for visualization, ease of use for driving the finite element models and for providing predefined data points for comparison with the model predictions. For passive flexion, data points were taken from indices corresponding to 5° flexion increments using desired kinematics data. For all other trials, data points were taken from indices where consecutive values were the same in the desired kinetics data. The highest index at which the values were the same were saved for plotting.  The disseminated data is provided in its raw form and Python scripts to perform the downsampling are available on the data site [1] in the ‘src’ folder.

The anterior-posterior laxity data from the repeatability tests were considered to quantify the repeatability of testing and possible relaxation of the joint throughout testing. The range of motion between the ±100 N anterior drawer extremes were calculated by averaging the last 0.25 s at the end of each hold (Table 5).

**Table 5:** Repeatability results for anterior-posterior laxity tests conducted during different time points of joint testing. Range of motion (ROM) between the ±100 N anterior drawer extremes were calculated by averaging the last 0.25 s at the end of each hold period where loading was sustained. ROM was reported for each of the three repeatability tests for all the tested specimens except specimen oks004. For oks004 posterior drawer was not a pure laxity test, hence it was excluded from this assessment.

	Anterior/Posterior range of motion (ROM), mm							
	oks001	oks002	oks003	oks004	oks006	oks007	oks008	oks009
Beginning	13.5	10.4	5.9	-	10.0	10.8	8.7	7.9
Middle	14.5	11.5	6.7	–	11.1	12.6	9.6	8.7
End	14.6	11.4	6.6	–	11.1	12.3	9.7	8.4

**Fig. 5:**
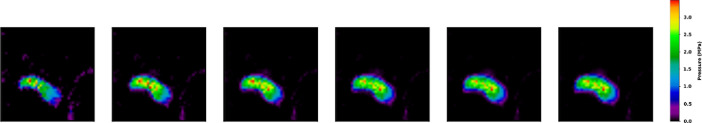
Patellofemoral pressure distribution for specimen oks003 at 60° flexion with quadriceps load applied at 100, 200, 300, 400, 500 and 600 N.

Contact pressure maps were obtained for each loading combination from the patellofemoral joint testing. Fig. 5 shows the pressure distribution for 60° flexion with 100 – 600 N quadriceps loads applied in 100 N increments for specimen oks003.

## 2 Experimental Design, Materials and Methods

### 2.1 Specimen preparation

Specimens were obtained from full legs, from the femoral head down to and including the foot. The soft tissue was dissected down to the beginning of diaphysis, but left intact around the region of the tibiofemoral and patellofemoral joints to preserve their in situ biomechanical environment and maintain hydration of the tissue during imaging and experimentation.

Computational modeling and simulation involves spatial registration of knee joint geometry, derived from anatomical images, with respect to the subsequent mechanical experimentation describing joint kinematics-kinetics. To accomplish this, rapid prototyped registration markers with internal spherical volumes containing an MRI-opaque background substance (solid MRI-transparent external material: VeroClear; MRI-opaque internal material: FullCure705; PolyJet 3D, Stratasys, Minnesota, USA) were rigidly fixed to the bones (femur, tibia, patella). To reduce metal induced imaging artifacts, brass screws were used to secure hardware needed for the motion capture system used in joint testing. For the larger bones, femur and tibia, separate spherical registration markers were developed with an outer diameter of 20 mm and three were affixed to each bone. Due to the smaller region for fixation on the patella, a separate rapid-prototyped triad containing three smaller spherical MRI-opaque regions (6 mm diameter) was fabricated to register patellar geometry (to be obtained from images) with experimental patella kinematics. The patella marker triad had 12 divot points and the dimensional relationship between the divots and spheres were known (and disseminated with data). The centers of these spheres were calculated from surface coordinates of the spherical geometry from MRI, and from mechanical testing (during which points on the outer spherical surface were digitized for femur and tibia markers and divots for patella marker assembly), to accurately align imaging and mechanical testing coordinate systems. An optoelectronic camera system (Optotrak, Northern Digital Inc., Waterloo, Canada) was used to track the motions of the bones during testing (tibia, femur and patella). Six degree of freedom (DoF) motion tracking optoelectronic orthopedic research pin markers were rigidly placed on each bone. The markers were positioned as close to the joint center as possible to minimize effects of bone bending and oriented in a way so they would be visible to the cameras throughout testing.

With the MRI registration spheres and the motion tracking marker assemblies properly secured to each bone, location of anatomical landmarks were collected. A custom optoelectronic probe was used to digitize anatomical landmarks and MRI registration spheres while the leg was fully intact. Ten points were collected on each sphere so that a sphere fit could be calculated, outputting sphere center and diameter. Tibia anatomical landmarks consisted of: T1. Medial tibial plateau (most medial point), T2. Lateral tibial plateau (most lateral point), T3. Medial malleolus of the tibia (most medial point), and T4. Lateral malleolus of the fibula (most lateral point). Femur anatomical landmarks consisted of: F1. Lateral epicondyle of the femur (most lateral point), F2. Medial epicondyle of the femur (most medial point), and F3-F6. 4 points around the epiphyseal line of the femur. Each probed point was collected along with a snapshot of the 6 DoF motion tracking marker attached to the respective bone. For the patellofemoral joint experimentation, the location of the 12 divot points on the registration marker assembly was acquired. To determine the centers of the spheres in the patella marker assembly, the dimensional relationship of the divots and centers of the spheres in the marker assembly was used. The location of the file describing this relationship is provided in the Data description section. Anatomical landmarks on patella were acquired at the most lateral, medial, superior and inferior points. The quadriceps line of action was also digitized at neutral joint state. The order of digitization was inferior point, center point, and superior point. As the registration marker assembly and the Optotrak marker for patella could not be placed at the same time on the patella, data were collected separately to associate Optotrak and registration marker coordinate systems. For this, an Optotrak marker and the patella base plug were placed on a piece of wood, followed by placing the patella Optotrak marker on the base plug. Location of Optotrak markers were recorded in Optotrak coordinate system. Patella Optotrak marker was replaced patella registration marker assembly on the patella base plug and location of Optotrak marker on wood and patella marker assembly divots were recorded. With these the transformation matrix between the patella registration marker coordinate system and Optotrak marker coordinate system could be established.

Once all the points were collected, the diaphyses of the femur and tibia were transected so that the overall length of the specimen was ∼380 mm, symmetric about the tibiofemoral joint (∼190.5 mm to either side). Bone cut lengths were determined to both retain relevant soft-tissue around the joint and to fit the specimen into the experimental joint testing system and a transportation tube for MRI.

### 2.2 MRI

Initial MRI settings to delineate each tissue type were adopted from the protocols of the Osteoarthitis Initiative (OAI) [5]. An iterative process of seven imaging trials were conducted to modify these initial protocols such that the final protocols produced images adequate for delineation of knee structures to be modeled. Five observers assessed the images for ease of deliniation before the protocols were finalized. A Siemens Skyra, 3.0 Tesla human MRI scanner (Siemens Healthneers Malvern, PA, USA) with a knee receiver coil (Tx/Rx CP Extremity Coil, Siemens Healthneers Malvern, PA, USA) was used for imaging. The receiver coil was 256 mm long with a 154 mm inner diameter. Additionally the goal was to balance image spatial resolution and tissue contrast while accommodating a total image acquisition time of less than 2 h. A resulting set of three different MRI protocols were chosen with the intention of identifying: 1) all structures and registration markers as a general-purpose reference, 2) cartilage and possibly menisci, and 3) connective tissue, including ligaments, tendons, and possibly menisci. The general-purpose imaging protocol was a 3D T1-weighted sequence without fat suppression, with an isotropic resolution of 0.5 × 0.5 × 0.5 mm (echo time (TE) = 6.01 ms, and repetition time (TR) = 20 ms). The cartilage imaging protocol was a 3D T1-weighted sequence with fat suppression, having an anisotropic resolution of 0.35 × 0.35 × 0.7 mm (TE = 5.34 ms, and TR = 29 ms. The connective tissue imaging protocol was a 2D proton-density, turbo spin echo sequence with an anisotropic resolution of 0.35 × 0.35 × 2.8 mm (TE = 9.7 ms, TR = 10,000 ms, 14 echoes). The connective tissue imaging protocol was acquired in the three standard anatomical orientations, with the high-resolution in-plane dimension (0.35 × 0.35 mm) lying in the axial, sagittal and coronal planes of the knee. Details of MRI sequence settings can be found in Table 6. The reference imaging coordinate systems remained the same for all three scanning protocols, which allows the components of the knee model, defined from the appropriate image sequence, to be easily combined spatially. Specimens were inserted femur first in the MRI machine (with the exception of oks002). MRI was obtained in the DICOM medical image format (https://www.dicomstandard.org/) and converted to NIfTI format for distribution (https://nifti.nimh.nih.gov/).

**Table 6:** MRI protocol specifications. Settings for all imaging protocols are provided in details.

Protocol ID	General Purpose	Cartilage	Tendon/Ligament
Protocol Type	3D, T1-weighted	3D, T1-weighted	Proton density, turbo spin echo
In-Plane Orientation(s)	3D isotropic (sagittal)	sagittal	axial, coronal, sagittal
Resolution (mm)	0.5 × 0.5 × 0.5	0.35 × 0.35 × 0.7	0.35 × 0.35 × 2.8
Fat Saturation	No	Yes	No
Matrix (phase)	316	448	432
Matrix (freq.)	480	512	512
Number of slices	320	224	50
Field of View [FOV] (mm)	158 × 240	157 × 180	151 × 180
Slice thickness/gap (mm/mm)	0.5/0.0	0.7/0.0	1.4/1.4
Flip angle (deg.)	25	25	90/150
TE/TR (ms/ms)	6.01/20	5.34/29	9.7/10,000
Bandwidth (Hz/pixel)	210	210	222
Chemical shift (pixels)	N/A	N/A	N/A
No. excitations averaged	1	1	1
Echo Train Length [ETL]	1	1	14
Phase encode axis	anterior-posterior	anterior-posterior	anterior-posterior
Distance factor (%)	N/A	N/A	100%
Phase oversampling	0	0	0
Slice oversampling	0	0	0
Phase resolution	0.5	0.35	0.35
Phase partial Fourier (8/8 = 1)	OFF	6/8	OFF
Readout partial Fourier (8/8 = 1)	OFF	OFF	OFF
Slice partial Fourier (8/8 = 1)	7/8	6/8	OFF
X-resolution (mm)	0.5	0.35	0.35
Y-resolution (mm)	0.5	0.35	0.39
Scan Time (min.)	21:18	27:18	4:52

### 2.3 Tibiofemoral joint testing

Once imaging was completed, the MRI registration markers were removed. The fibula was secured to the tibia by pre-drilling through the fibula and tibia and driving a screw through both bones as close to the joint center as possible. Efforts were made to maintain anatomical relative positions between the two bones. The fibula was cut off just distal to the screw.

Both the tibia and femur ends were rigidly secured to 76.2 mm long and 63.5 mm diameter aluminum tubes. Each bone was potted individually. The end of the bone was placed down into the center of the tube. Melted Wood's metal was poured into the tube and the bone was held in place until the metal hardened. Efforts were made prior to potting to ensure the 6 DoF motion tracking markers could be secured with the pot in place. Efforts were also made to ensure the total end-to-end length was 381 mm after potting and that the two metal tubes were close to be in line with each other when the knee was at full extension. Two drill bits were cross drilled into each tube to prevent spinning or any pulling out of the Wood's metal in the tube. They were drilled towards the end of the tube closest to the joint center.

Testing was performed on a 6 DoF parallel robot (Rotopod R-2000, Mikrolar, Hampton, NH) with a custom rotary stage mounted to the top to yield a 7th DoF for increased range of motion. Loads were measured with a 6-axis universal force sensor (UFS) (Theta, ATI, Apex, NC) rigidly attached to the frame. Joint kinematics and kinetics were controlled and measured using simVITRO® software (simVITRO, Cleveland Clinic, Cleveland, OH) which was developed using LabVIEW (National Instruments, Austin, TX). Prior to testing, the robot and UFS coordinate systems were registered in the software by collecting points using the optoelectronic probe and establishing spatial relationships. The UFS was tared and general estimations of the potted tibia mass (13–22 N) and center of mass (75 mm) was accounted for. The tibia pot was then mounted to a fixture rigidly attached to the UFS and the robot was driven so the femur pot could be mounted to it with the knee extended. Once the specimen was attached, the robot position was fine-tuned using the simVITRO® software to achieve close to zero loads on the UFS. Details of the procedure can be found in simVITRO® testing setup tutorial disseminated at the data site [1] in the ‘doc’ folder. This location was considered the neutral position of the knee. With the motion tracking markers secured to each bone, a snap shot was taken of each 6 DoF motion tracking marker and the robot position. Using the spatial relationship between the anatomical points of each bone and the respective 6 DoF motion tracking marker, coordinate systems and spatial relationships between the hardware and the specimen were established. These spatial relationships allowed the joint coordinate system (JCS) of the knee (see documentation on knee coordinate systems at the data site [1] in the ‘doc’ folder), as an adaptation of Grood and Suntay convention [6], to be calculated with sensors in two different ways; 1) using robot position and 2) using the 6 DoF optoelectronic motion tracking sensors. Tibia loads were measured by transforming loads measured by the UFS to the tibia coordinate system and reporting them as loads applied to tibia in a clinically relevant manner.

An optimized version of the knee joint coordinate system, which attempted to find the functional mechanical axis, was established before proceeding the subsequent testing. The tibiofemoral joint underwent passive flexion from 0° to 90° with a 50 N compression load. The position and orientation of the femur coordinate system was optimized to minimize the off-axis translations and rotations during the passive flexion motion. This process is intended to isolate the functional mechanical axis of the tibiofemoral joint. Due to this approach the screw home mechanism, i.e. off-axis rotations, may be minimized. Once the optimized coordinate system had been established, the knee was fully extended and positioned in a zero loaded state so that the neutral position could be re-established. Passive flexion was performed again to verify that off-axis motions were decreased.

The tibiofemoral joint underwent preconditioning where the joint was positioned at 30° flexion and laxity loads of ± 5 Nm internal rotation (IR) torque, ± 10 Nm varus torque, and ±100 N anterior drawer were applied sequentially on the tibia, in the tibia fixed coordinate system. Testing protocols were adapted from Borotikar [7]. After pre-conditioning, two general types of tests were performed; laxity loading and combined loading. Both testing types were performed at 0°, 30°, 60° and 90° flexion and a 20 N compression load was applied throughout testing. The laxity loading consisted of incrementally ramping and holding internal/external rotation torque, varus/valgus torque and anterior/posterior force sequentially: 1) Ranges of 0 to ± 5 Nm of IR torque was applied at increments of 1 Nm with 10 second holds after each incremental increase. 2) Ranges of 0 to ± 10 Nm of varus torque was applied at increments of 2.5 Nm with 10 second holds after each incremental increase. 3) Ranges of 0 to ± 100 N of anterior drawer force was applied at increments of 10 N with 10 second holds after each incremental increase. The combined loading consisted of all permutations consisting of IR torque of −5, 0, and 5 Nm, varus torque of −10, 0, and 10 Nm and anterior drawer of −100, 0, and 100 N. For each permutation, a 12 second hold was maintained. A repeatability test was performed three times throughout testing; (1) beginning, after preconditioning, (2) middle, prior to flexing to 60°, and (3) end, after completing all testing. The repeatability test was an anterior drawer laxity test (0 to ± 100 N applied at increments of 10 N with 5 second holds) performed at 30° flexion.

### 2.4 Patellofemoral joint testing

The patellofemoral joint coordinate system adopted Grood and Suntay convention [6] for description of the patella-femoral kinematics. The anatomical landmarks for the patella coordinate system matched those recommended by Kedgley et al. [8]. To measure the patellofemoral contact mechanics (contact pressure distribution, area, and total force) a Tekscan (Boston, MA) pressure sensor (5051, 8.27 MPa range) was utilized. The sensor was plugged into a computer running Tekscan software (version 7.6). To protect the sensor from the environment of the joint it was sealed using a lamination sheet. A two-step equivalencing and calibration process was completed. Two-point equivalencing was performed using a pressure bladder system to evenly load the Tekscan sensor at 300 kPa and 600 kPa. A 5 point calibration was completed using an Instron 8511 (Instron, Norwood MA) test frame with loads of 50, 100, 200, 400, and 600 N. The sensor was supported by a flat aluminum plate with 4 sheets of 0.3 mm thick rubber and the Instron compressed the sensor with a baseball as an indenter. The manufacturer recommended that the sensor be loaded using items of similar stiffness and geometry as a patellofemoral joint and the baseball and rubber topped plate were selected as an approximation of these conditions. Following joint level testing in the robot the sensor was returned back to the calibration setup and the calibration process was repeated. Two calibration files (before and after) were created in order to bound the potential uncertainty in the pressure measurements.

The specimen was then secured to the 6 DoF parallel robot. The tibia was secured to a custom stage rigidly attached to the robot. Loads were measured with a custom sensor composed of two 6 DoF force-torque sensors (SI-1900–80, ATI Industrial Automation, Apex, NC) embedded in stage. Sensor loads were transformed to tibia loads and were used to provide feedback needed to drive the robot. The femur was mounted pointing upwards, attached to the stationary frame and the tibia was attached to the moving frame through the load transducer. The reason tibia was up in tibiofemoral joint testing is because loads were measured on the tibia and it was more desirable to have a static load cell mounted directly to the tibia to avoid dealing with changing gravitational effects or inertial effects of the load cell moving. This could not be done for patellofemoral joint testing because, the femur needed to be mounted upwards so that the quad actuator could pull on it, and the static load cell mounted to the femur could not be used because it would be measuring the effects of the in situ loads and quad loads together which was not desired. Gravity compensation of the calculated tibia loading was provided by simVITRO®. A quadriceps loading system was developed utilizing a Baldor (Fort Smith, AR) model BSM80N-275AE servomotor and a harmonic drive system (CSG-40–50, Hauppauge, NY). The quadriceps tendon was held by a custom wire mesh grip (DCD Design and Manufacturing Ltd., Richmond BC, Canada) which was further secured to the tendon by freezing the tendon-grip interface with liquid nitrogen. Quadriceps line of action was set to approximate the sulcus defined (inferior-superior) direction of the trochlear groove and was measured relative to the femoral coordinate system. Quadriceps loading was applied under force feedback control synchronized with the robot to achieve a desired joint loading state. The Tekscan sensor was inserted in the patellofemoral joint through the opening under the quadriceps tendon to measure contact mechanics.

A kinetics based neutral position (minimal loading) was established once the femur was set to 0° flexion angle by operating the robot in force control mode similar to the tibiofemoral joint testing. Then, the tibiofemoral joint was moved through a passive flexion motion from 0° to 60° flexion with a 50 N compressive load. The femur coordinate system was then optimized to minimize the change in joint translations and off-axis rotations throughout the flexion cycle. This process was used to define a functional mechanical axis of the femur similar to the tibiofemoral joint testing. Following the optimization, the kinetics based neutral position was re-established with the new coordinate system. Patellofemoral mechanics and kinematics were characterized under quadriceps loading at tibiofemoral flexion angles of 0°, 15°, 30°, 45°, and 60°. At each flexion angle the tibiofemoral joint was set to a position approximating passive flexion by minimizing all off axis loads except a 20 N tibiofemoral compression. Then, the tibiofemoral kinematics were fixed and the quadriceps loads were applied using the following increments: 20, 100, 200, 300, 400, 500, and 600 N. At each loading state, the patellofemoral contact mechanics, patellofemoral kinematics, tibiofemoral kinematics, and tibial loads were measured for approximately 5 s. At each flexion angle at 20 N quadriceps load, patellofemoral contact patch was verified to be approximately at the center of the sensor.

### 2.5 Coordinate system considerations for joint mechanical testing

Data acquisition system, simVITRO®, utilizes “right knee abstraction” for control and data collection so that the data from multiple specimens can easily be combined regardless of whether or not the specimen was actually from the right or left side. However, for specimen specific modeling and combining kinematic data with imaging data sets, the data, including kinematics-kinetics information and coordinate transformation matrices may need to be transformed back to “physical representation” for the left knees to achieve appropriate description in the physical space. The transformations are mirroring operation. Further details are available in the documentation on knee coordinate systems provided on the data site [1] available in the ‘doc’ folder.

## Ethics Statement

(i) The specimens were from cadaver donors.  They were acquired through accredited specimen suppliers (Science Care, Inc. and Anatomy Gifts Registry), who consented donors for the use of donated tissue for education and training, scientific advancement, and/or research and development purposes.
(ii) According to U.S. federal regulations (45 CFR Part 46), the study did not qualify as research involving human subjects. The project proposal was submitted to the funding agency and approved for funding under these circumstances.
(iii) No individually identifiable health information about the donors was disseminated. Demographics information is provided to support research utility of the specimens. None of the donors belong to any rare identifiable group.

## Credit author statement

**Snehal Chokhandre:** Workflow Conceptualization, Methodology development, Data curation and dissemination, Writing; **Erica E. Neumann:** Data analysis codes

**Tara F. Nagle:** Mechanical testing conceptualization, Methodology development, Writing; **Robb W. Colbrunn:** Mechanical testing conceptualization, Methodology development, Writing; **Chris A. Flask:** Imaging methodology; **Ceylan Colak:** Tissue quality evaluation; **Jason Halloran:** Conceptualization, Methodology, Writing; **Ahmet Erdemir:** Study conceptualization, Supervision. All authors reviewed the manuscript.

## Declaration of Competing Interest

Robb W. Colbrunn and Tara F. Nagle receive royalties from each copy of simVITRO® software sold under a licensing agreement from the Cleveland Clinic Innovations. Ahmet Erdemir owns and operates innodof, LLC, a consulting company for modeling and simulation. The remainder of author(s) declare no competing interests.

## Appendix Supplementary materials

Image, application 1
