# Supplementary material for: Specimen specific imaging and joint mechanical testing data for next generation virtual knees
Source: Data Brief. 2021 Jan 30;35:106824. doi: 10.1016/j.dib.2021.106824 (PMC7890148; doi:10.1016/j.dib.2021.106824)
Supplement: Supplementary file 1 [file mmc1.docx]

Appendix

A1. Joint mechanical testing data folder descriptions.

joints_mechanics_oks###.zip
.
|
|--PatellofemoralJoint
| |
| |--ContactPressure
| | |
| | |-...
| | |
| | |-*.cal - Sensor calibration files
| | |-*.equ - Sensor equilibration files
| | |-*.csv - Contact pressure data at various loading states (in human readable form)
| | |-*.fsx - Contact pressure data at various loading states (in binary form)
| | |
| | |-...
| |
| |--KinematicsKinetics
| |
| |-...
| |
| |-0*_*_* - Experimentation trials folders for various loading states
| | |
| | |-Configuration
| | | |
| | | |-*Project.sVprj - copy of robotics testing configuration
| | |
| | |-Data
| | |
| | |-...
| | |
| | |-*Main.tdms - raw kinematics/kinetics data
| | |-*main_processed.tdms - time synchronized and interpolated kinematics/kinetics data
| | |
| | |-...
| |
| |-Configuration - main robotics testing configuration
| | |
| | |-...
| | |
| | |-*Project.sVprj - main robotics testing configuration
| | |-*Quad*LOA*.cfg - quadriceps line of action
| | |
| | |-...
| |
| |--...
|
|--TibiofemoralJoint
| |
| |--KinematicsKinetics
| |
| |-...
| |
| |-0*_*_* - Experimentation/reproducibility trials folders for various loading states
| | |
| | |-Configuration
| | | |
| | | |-*Project.sVprj - copy of robotics testing configuration
| | |
| | |-Data
| | |
| | |-...
| | |
| | |-*Main.tdms - raw kinematics/kinetics data
| | |-*main_processed.tdms - time synchronized and interpolated kinematics/kinetics data
| | |
| | |-...
| |
| |-Configuration - main robotics testing configuration
| | |
| | |-...
| | |
| | |-Patella* - patella registration data
| | | |
| | | |-...
| | | |
| | | |-CAD_PT_DIMENSIONS.txt - points patella registration marker assembly in CAD
| | | |-*.tdms - relative pose and orientation of patella and reference motion analysis marker sets
| | | |-State.cfg - configuration file including probed points on patella registration marker assembly
| | | |
| | | |-...
| | |
| | |-*Project.sVprj - main robotics testing configuration
| | |
| | |-...
| |
| |--...
|
|--readme.txt - this file


(1) Joints tested -- The joint mechanics testing folder contains data for both patellofemoral joint testing and for tibiofemoral joint testing.

(2) Patellofemoral joint contact pressures -- Contact pressures are provided for the patellofemoral joint only.

(2-a) Patellofemoral joint contact pressures - calibration files -- Contact pressures were measured by a sensor equilibrated calibrated both pre- and post mechanical joint testing. Date of equilibration and calibration are appended to the filenames (pre-experiment calibration with the earlier date); file extension are (.equ) for equilibration and (.cal) for calibration. Ideally, both calibrations should be the same, indicating sensor being intact throughout the test.

(2-b) Patellofemoral joint contact pressures - experiment trials files -- Data were collected and stored in original Tekscan proprietary format (.fsx) and were exported in human readable text format (.csv). .fsx files can be opened in proprietary Tekscan software to apply any desirable calibration; .csv files are exported using one of the calibration files. Trial filenames include the specimen number, joint condition, flexion angle in degrees, quadriceps force in Newtons and the trial number. These should match with the patellofemoral joint kinematics kinetics trial data folders, which were collected simultaneously. Additional files with various descriptive names may exist.

(2-c) Patellofemoral joint contact pressures - sensor directionality -- Various data files indicate the directionality of the sensor, i.e. those with filenames appended with "lateral", "medial", "superior", "inferior".

(3) Patellofemoral joint kinematics/kinetics -- Patellofemoral joint kinematics/kinetics data are provided for different levels of quadriceps loading under various flexion angles.

(3-a) Patellofemoral joint kinematics/kinetics - experiment trials folders -- Trial folder names include the trial number, flexion angle in degrees and quadriceps load in Newtons. These should match with the patellofemoral joint contact pressures trial files, which were collected simultaneously. Each trial folder includes "Configuration" and "Data" subfolders. Additional folders with various descriptive names may exist, e.g. for passive flexion of the tibiofemoral joint, pre-conditioning runs, etc. Some experiment trial folders may be empty, may not contain any data in subfolders, or may have partial data, i.e., without post-processing. These are mostly aborted trials. They are provided in here simply to illustrate the loading history of the specimen. As a result, it is likely that desired experimentation conditions may be duplicated in folder names. It is advised to utilize data folders containing post-processed data files, i.e. those with *main_processed.tdms (see note (3-a-2) below).

(3-a-1) Patellofemoral joint kinematics/kinetics - experiment trials folders - configuration -- "Configuration" folder contains a Project.sVprj, which is a configuration file generated and used by the robotics testing system. This is a compressed file which can be uncompressed, e.g. using unzip, to access to important files such as the State.cfg. These files should contain abridged copies of main configuration file (see note (3-b) below).

(3-a-2) Patellofemoral joint kinematics/kinetics - experiment trials folders - data -- Kinematics/kinetics data are in the "Data" folder within files with the extension .tdms. These are binary files, which can be accessed by Python packages such as free and open source npTDMS. *Main.tdms contains the raw data *main_processed.tdms contains time synchronized and interpolated data (a good starting point to explore data). For left knees, additional .tdms files are included. This is a result of experimentation system using right knee abstraction, therefore *Main.tdms and *main_processed.tdms containing data in the right knee abstraction. Additional processing converts such data to physical representation for the left knees, appropriate for prospective registration with imaging data and therefore model development. For more detailed information on the contents of all these files are provided as part of the documentation on the robotics testing system at http://wiki.simtk.org/openknee/Infrastructure/ExperimentationMechanics.

(3-b) Patellofemoral joint kinematics/kinetics - main configuration folder -- This folder includes files for patellofemoral joint testing configuration and within, auxiliary data including anatomical landmarks, registration markers, coordinate systems, reference kinematics/kinetics states of the joints, etc., all in human readable text file formats. The user is encouraged to refer to main tibiofemoral joint configuration folder contents (see note (4-c) below) for data on patella registration marker assembly.

(3-b-1) Patellofemoral joint kinematics/kinetics - main configuration folder - project file -- *Project.sVprj is the main configuration file generated and used by the robotics testing system. This is a compressed file which can be uncompressed, e.g. using unzip, to access to important files such as the State.cfg. This file contains anatomical landmarks on patella, femur, and tibia and configuration of motion analysis markers.

(3-b-2) Patellofemoral joint kinematics/kinetics - main configuration folder - quadriceps line of action -- A file indicating in its name the quadriceps loading and line of action (LOA) contains at least three points probed along the cable and freeze clamp system used to apply forces to the patella.

(4) Tibiofemoral joint kinematics/kinetics -- Tibiofemoral joint kinematics/kinetics data are provided for different loading conditions including laxity tests and combined loading scenarios.

(4-a) Tibiofemoral joint kinematics/kinetics - experiment trials folders -- Trial folder names include the trial number, loading condition and flexion angle in degrees. Additional folders with various descriptive names may exist, e.g. for passive flexion of the tibiofemoral joint, pre-conditioning runs, etc. Some experiment trial folders may be empty, may not contain any data in subfolders, or may have partial data, i.e., without post-processing. These are mostly aborted trials. They are provided in here simply to illustrate the loading history of the specimen. As a result, it is likely that desired experimentation conditions may be duplicated in folder names. It is advised to utilize data folders containing post-processed data files, i.e. those with *main_processed.tdms (see note (4-a-2) below).

(4-a-1) Tibiofemoral joint kinematics/kinetics - experiment trials folders - configuration -- "Configuration" folder contains a Project.sVprj, which is a configuration file generated and used by the robotics testing system. This is a compressed file which can be uncompressed, e.g. using unzip, to access to important files such as the State.cfg. These files should contain abridged copies of main configuration file (see note (4-c) below).

(4-a-2) Tibiofemoral joint kinematics/kinetics - experiment trials folders - data -- Kinematics/kinetics data are in the "Data" folder within files with the extension .tdms. These are binary files, which can be accessed by Python packages such as free and open source npTDMS. *Main.tdms contains the raw data *main_processed.tdms contains time synchronized and interpolated data (a good starting point to explore data). For left knees, additional .tdms files are included. This is a result of experimentation system using right knee abstraction, therefore *Main.tdms and *main_processed.tdms

containing data in the right knee abstraction. Additional processing converts such data to physical representation for the left knees, appropriate for prospective registration with imaging data and therefore model development. For more detailed information on the contents of all these files are provided as part of the documentation on the robotics testing system at http://wiki.simtk.org/openknee/Infrastructure/ExperimentationMechanics.

(4-b) Tibiofemoral joint kinematics/kinetics - reproducibility trials folders -- At three time points during the experimentation, anterior-posterior laxity testing was repeated to assess reproducibility of the joint mechanical response. Folder naming convention (including referral to anterior-posterior (AP) laxity) and folder contents are similar to those of the experiment trials folders. The lowest trial number refers to data collected at the beginning, the highest trial number refers to data collected at the end, the middle refers to data collected at the mid-time point. With the assumption that the same loading conditions (kinetics profile) are applied, it is anticipated that the movement of the tibiofemoral joint will be the same. Any discrepancies may be attributed to damage to the joint or conditioning effects.

(4-c) Tibiofemoral joint kinematics/kinetics - main configuration folder -- This folder includes files for tibiofemoral joint testing configuration and within, auxiliary data including anatomical landmarks, registration markers, coordinate systems, reference kinematics/kinetics states of the joints, etc., all in human readable text file formats. A folder for patella registration also exists as landmark collection on patella registration markers were acquired before tibiofemoral joint testing started. Other folders auxiliary to experimentation may exist.

(4-c-1) Tibiofemoral joint kinematics/kinetics - main configuration folder - project file -- *Project.sVprj is the main configuration file generated and used by the robotics testing system. This is a compressed file which can be uncompressed, e.g. using unzip, to access to important files such as the State.cfg. This file contains anatomical landmarks on femur, tibia, and patella, registration markers on femur and tibia, and configuration of motion analysis markers.

(4-c-2) Tibiofemoral joint kinematics/kinetics - main configuration folder - patella registration -- A folder indicating in its name the patella registration contains data to properly align patella registration spheres and probed divot points on patella registration markers. "State.cfg" contains divot points probed by motion analysis system relative to an reference motion analysis marker set. A .tdms file, potentially referring to registration marker relative placement, contains relative pose and orientation of the local patella motion analysis marker set relative to the reference motion analysis marker set. "CAD_PT_DIMENSIONS.txt" contains location of patella registration spheres and divots in the CAD coordinate system.

(5) Additional information -- Various details relevant to joint mechanics testing can be found at various wiki pages at the project site.

(5-a) Additional information -- For specimen-specific protocol deviations and experimentation notes, see
http://wiki.simtk.org/openknee/oks001

(5-b) Additional information -- For documentation on experimental protocols, see
http://wiki.simtk.org/openknee/Specifications/SpecimenPreparation
http://wiki.simtk.org/openknee/Specifications/ExperimentationJointMechanics

(5-c) Additional information -- For documentation on robotics testing system including folder organizations and file contents, see
<http://wiki.simtk.org/openknee/Infrastructure/ExperimentationMechanics>

A2. Joint mechanical testing file contents.

Table A1. Data file descriptions. Details for each channel included in the joint testing data file are provided.

| **Group** | **Channel(s)** | **Notes** |
| --- | --- | --- |
| Attributes | Version | This is the simVITRO version of the data file and can be used for proper version control and data interpretation for automated post-processing of the file contents. |
| Kinematics.JCS.Desired | Medial Translation - Desired Posterior Translation - Desired Superior Translation - Desired Flexion Angle - Desired Valgus Angle - Desired Internal Rotation Angle - Desired | This is the desired kinematic state of the tibiofemoral joint. More specifically, it is the kinematic setpoint that the controller was being given, at the time instant this data was recorded to the file, as an input to the controller. |
| Kinematics.JCS.Actual | Medial Translation Posterior Translation Superior Translation Flexion Angle Valgus Angle Internal Rotation Angle | This is the kinematic state of the tibiofemoral joint. More specifically, it is the position that the joint was commanded to be at for the time point this data was recorded in the file. Depending on the speed of the system at the time this may or may not be a perfect representation of the actual joint position. These kinematics are relative JCS kinematics, not absolute JCS kinematics. The offset between the two can be found in the State configuration file in the 'JCS' state section. In addition, if the femur coordinate system was optimized as a result of the experiment setup, this state will reflect the kinematics given the new, optimized, coordinate system. |
| State.JCS | JCS Medial JCS Posterior JCS Superior JCS Flexion JCS Valgus JCS Internal Rotation | This is the kinematic state of the tibiofemoral joint. More specifically, it is the position that the joint is calculated to be given the most recently measured robot position. Compared to the Kinematics.JCS.Actual group, this group is likely to be a better representation of the actual joint position. However, it is subject to some temporal uncertainty of measuring the robot position. These kinematics are relative JCS kinematics, not absolute JCS kinematics. The offset between the two can be found in the State configuration file in the 'JCS' state section. In addition, if the femur coordinate system was optimized as a result of the experiment setup, this state will reflect the kinematics given the new, optimized, coordinate system. |
| State.Knee JCS | Knee JCS Medial Knee JCS Posterior Knee JCS Superior Knee JCS Flexion Knee JCS Valgus Knee JCS Internal Rotation | This is the kinematic state of the tibiofemoral joint. More specifically, it is the position that the joint is calculated to be given the most recently measured positions of the motion capture sensors. Compared to the Kinematics.JCS.Actual group, this group is likely to be a better representation of the actual joint position. However, it is subject to some temporal uncertainty of measuring the motion tracking marker position. Compared to the State.JCS group, this group is likely to be a better representation of the actual joint position in that it can account for bone bending artifacts (depending on where the sensors are mounted). However, it is likely to have a larger noise floor compared to the JCS calculations based on robot position. These kinematics are relative JCS kinematics, not absolute JCS kinematics. The offset between the two can be found in the State configuration file in the 'Knee JCS' state section. In addition, if the femur coordinate system was optimized as a result of the experiment setup, this state will NOT reflect the kinematics given the new, optimized, coordinate system. It will reflect only the original digitized coordinate system. |
| Kinetics.JCS.Desired | Lateral Drawer - Desired Anterior Drawer - Desired Distraction - Desired Extension Torque - Desired Varus Torque - Desired External Rotation Torque - Desired | This is the desired kinetic state of the joint (i.e. tibia loads). More specifically, it is the kinetic setpoint that the controller was being given, at the time instant this data was recorded to the file, as an input to the controller. |
| State.JCS Load | JCS Load Lateral Drawer JCS Load Anterior Drawer JCS Load Distraction JCS Load Extension Torque JCS Load Varus Torque JCS Load External Rotation Torque | This is the kinetic state of the joint (i.e. tibia loads). More specifically, it is the joint (tibia) loads that are calculated given the most recently measured load cell output and the relative position of the tibia and load cell from the previous loop. Depending on the mounting configuration, and the speed of the system at the time, the temporal uncertainty in the relative position may or may not affect the computed tibia loads. For example, if the tibia is statically mounted to the load cell, then it has no influence on the measurement. If the load cell is mounted to the femur and the system is moving slowly, then again it will have little to no influence. It is only in the case where the femur is mounted to the load cell, and the system is moving quickly, that a chance for the temporal uncertainty to have any affect is possible. |
| Kinematics.Robot.Actual | x y z roll pitch yaw | This is the robot position. More specifically, it is the position that the robot was commanded to be at for the time point this data was recorded in the file. Depending on the speed of the robot at the time this may or may not be a perfect representation of the actual robot position. |
| Sensor.Robot Position | Robot Position_x Robot Position_y Robot Position_z Robot Position_roll Robot Position_pitch Robot Position_yaw | This is the most recently measured position of the robot in the native robot (WORLD2) reference frame. |
| Sensor.Femur Sensor | Femur Sensor_Femur.x Femur Sensor_Femur.y Femur Sensor_Femur.z Femur Sensor_Femur.r Femur Sensor_Femur.p Femur Sensor_Femur.w | This is the most recently measured position of the motion tracking sensor in the femur, in the native (WORLD1) reference frame. |
| Sensor.Tibia Sensor | Tibia Sensor_Tibia.x Tibia Sensor_Tibia.y Tibia Sensor_Tibia.z Tibia Sensor_Tibia.r Tibia Sensor_Tibia.p Tibia Sensor_Tibia.w | This is the most recently measured position of the motion tracking sensor in the tibia, in the native (WORLD1) reference frame. |
| Sensor.Static Load Cell  (Tibiofemoral testing only) | Static Load Cell_Fx Static Load Cell_Fy Static Load Cell_Fz Static Load Cell_Mx Static Load Cell_My Static Load Cell_Mz | This is the most recently measured loads from the load cell in the native load cell reference frame. |
| Kinematics.Nominal JCS Position | Untitled | The joint nominal position values are a 6x1 array of numbers that are the Nominal Position values on the simVITRO main screen. These values get written to the data file at the end of the experiment run. At any point throughout the experiment run the user can press the Define Nominal Position button to define that particular kinematic position as the Nominal Position. This value would be useful in the data file in the case where the user wants to store information about a reference position interesting to that particular experiment run. For example, if the kinematic values of the joint are non-zero under some nominal load state (or at a fixed flexion angle) and they want to measure the change in position, from this nominal position, when applying loads, then storing the nominal position can make quick work of post processing and calculating relative positions. |
| Timing.Control Loop Actual dt | Actual dt | This is the time between the the current and previous data acquisition loop. This loop performs, among other things, 1) data aqcuisition of digitized sensors, 2) state calculations, and 3) writing all data to disk. Note that this group name is a bit of a misnomer in that the control loop is separate from the data acquisition loop, and this value refers to the data acquisition loop. This data is used for resampling and time aligning the digitized sensor, and state data to have a constant dt in the main_processed file. |
| Timing.Sync Trigger | Setpoint Time | This is the time that the setpoint is passing to the controller to define trajectory execution timing. This data is used for time aligning the digitized sensor and state data to properly truncate the data before and after the actual trajectory execution. Note that in the main file this channel will have values of -1 before and after trajectory execution. In the main_processed file all values will be greater than or equal to zero. |
| Timing.Velocity Multiplier | Velocity Multiplier | This is the value of the master gain (a.k.a. blue slider that is treated as the system Sensitivity or Velocity Multiplier) at the time instant this data was recorded to the file. This data is used for iterative learning of trajectory gains by scaling the stored trajectory gains by the time history of this user controlled master gain. These values are in units of percentage where 100 represents an unmodified gain in the stored trajectory gain values. A value of 50 represents a reduction in the gain by one half. |

Table A2. Configuration file description. Details of all the configuration files are provided.

| Configuration File Name | Description |
| --- | --- |
| Actuator.cfg | Includes properties related to the robot  -Limits  -Velocity/Acceleration  -Spatial Relationships  -etc. |
| Project.cfg | Includes general project information, including:  -Data Acquisition Properties (filtering, sampling rate, etc.)  -Force Control Limits  -Max Motion Limits  -etc. |
| Sensor.cfg | Includes properties relating to the following sensors:  -Static Load Cell  -Digitizer  -Robot Position  -Femur Optoelectronic Sensor  -Tibia Optoelectronic Sensor |
| Setup.cfg | Includes properties relating to the following setup steps:  -Recording specimen information  -Initializing Hardware  -Defining the Robot Coordinate System  -Zeroing the Load cell  -Mounting the Specimen  -Defining the Rigid body coordinate system  -Determining Neutral Position |
| State.cfg | -Includes properties related to state relationships  -This file will include the digitized points and transformation matrices used to calculate coordinate systems and spatial relationships between hardware and the specimen |
| Trajectory.trj | Includes trajectory details in the form of setpoints throughout times  Trajectory elements are linearly interpolated between setpoints  Setpoints include:  -Kinematics  -Kinetics  -Hybrid Values (1: Force/Torque Control, 0: Position Control)  -Gains |

A3. Data organization at data site, [https://doi.org/10.18735/4e78-1311](http://archive.simtk.org/oks/MRI_JointMech/)

joint_mechanics

.

| -- joint_mechanics-oks00x.zip (for details see A1.)

Folder contains all mechanical testing data for both tibiofemoral and patellofemoral joints for each specimen. Each .zip folder contains a readme.txt with details of the subfolders and files.

mri

.

| -- mri-oks00x

Folder contains magnetic resonance imaging data for each specimen.

MRI protocols: 1. General purpose imaging: 3D T1-weighted without fat suppression, isotropic, 0.5 mm resolution. 2. Cartilage imaging: 3D T1-weighted with fat suppression, 0.35 mm sagittal plane resolution, 0.7 mm out of plane resolution. 3. Connective tissue imaging: Proton density, turbo spin echo in sagittal, axial, and coronal planes, 0.35 mm in plane and 2.8 mm out of plane resolution.

doc

Folder contains, 1. Documents to aid understanding of how the various tools required during mechanical testing are calibrated, used, programmed and controlled. 2. Document detailing notes taken on testing day and, any deviations from predefined specifications. 3. Static copies of project web pages as of date of upload.

src

Folder contains Python scripts to - 1. Generate transformation matrices necessary to perform patella, tibia and femur registration and calculate registration errors. 2. Resample and plot the mechanical testing data and visualize the trial. 3. Process the pressure data for visualization and extraction of necessary metrics.
